# Long-term health-related quality of life after trauma with and without traumatic brain injury: a prospective cohort study

**DOI:** 10.1038/s41598-023-30082-4

**Published:** 2023-02-20

**Authors:** Olivia Kiwanuka, Philipp Lassarén, Eric P. Thelin, Anders Hånell, Gabriel Sandblom, Ami Fagerdahl, Lennart Boström

**Affiliations:** 1 Department of Clinical Neuroscience, Karolinska Institute, Stockholm, Sweden; 2 Department of Surgery, Södersjukhuset, Stockholm, Sweden; 3 Department of Neurology, Karolinska University Hospital, Stockholm, Sweden; 4 Department of Clinical Science and Education, Södersjukhuset, Karolinska Institute, Stockholm, Sweden; 5 Department of Medical Sciences, Neurosurgery, Uppsala University, Uppsala, Sweden

**Keywords:** Quality of life, Trauma

## Abstract

To purpose was to assess and compare the health-related quality of life (HRQoL) and risk of depression two years after trauma, between patients with and without traumatic brain injury (TBI) in a mixed Swedish trauma cohort. In this prospective cohort study, TBI and non-TBI trauma patients included in the Swedish Trauma registry 2019 at a level II trauma center in Stockholm, Sweden, were contacted two years after admission. HRQoL was assessed with RAND-36 and EQ-5D-3L, and depression with Montgomery Åsberg depression Rating Scale self-report (MADRS-S). Abbreviated Injury Score (AIS) head was used to grade TBI severity, and American Society of Anesthesiologists (ASA) score was used to assess comorbidities. Data were compared using Chi-squared test, Mann Whitney U test and ordered logistic regression, and Bonferroni correction was applied. A total of 170 of 737 eligible patients were included. TBI was associated with higher scores in 5/8 domains of RAND-36 and 3/5 domains of EQ-5D (p < 0.05). No significant difference in MADRS-S. An AIS (head) of three or higher was associated with lower scores in five domains of RAND-36 and two domains of EQ-5D but not for MADRS-S. An ASA-score of three was associated with lower scores in all domains of both RAND-36 (p < 0.05, except mental health) and EQ-5D (p < 0.001, except anxiety/depression), but not for MADRS-S. In conclusion, patients without TBI reported a lower HRQoL than TBI patients two years after trauma. TBI severity assessed according to AIS (head) was associated with HRQoL, and ASA-score was found to be a predictor of HRQoL, emphasizing the importance of considering pre-injury health status when assessing outcomes in TBI patients.

## Introduction

Traumatic injuries in general, and traumatic brain injuries (TBIs) specifically, are the leading causes of death and disability among young people and account for 10% of all years lived with disability^1,2^). With improvements in acute care and management, more patients survive the initial phase, but traumatic injuries still account for a significant cause of hospitalization^3^.

There is an increasing awareness and interest in the perceived health related quality of life (HRQOL) as an outcome measure after trauma, including TBI^1,4,5^. Previous studies have shown that even a single incident of TBI may cause debilitation and impaired HRQOL compared to the general population^1,4–6^. Multiple studies also report a worsened HRQOL after trauma in general, with long-lasting pain and anxiety^7–11^. There are, however, conflicting results regarding the impact of injury severity on HRQOL. Some studies show a decreased HRQOL in severe TBI^12,13^, while others do not^14–16^, and some report a complex or even inverse relationship^17,18^. Concomitant TBI and polytrauma are associated with impaired HRQOL; polytrauma-patients with TBI have been shown to fare worse than polytrauma patients without^19^, and one study shows that TBI-patients with multiple extracranial injuries fare worse than patients with isolated TBI^20^. To the best of our knowledge, no study has compared HRQOL of trauma patients with regard to TBI in a mixed cohort. Many studies focus on the severe range of trauma, and there is a large knowledge gap regarding HRQoL after milder forms of TBI and trauma, which this study aims to bridge.

Depression and other psychiatric disorders are common following TBI^21–25^. Post-traumatic changes, such as neuroinflammation and neuroendocrine dysregulation are suggested to play an important role in the development of depression^26^. Choi et al.^27^ showed a long-term higher incidence of depression in TBI, compared to a control with other hospital encounters. Depression is also common after trauma without TBI^28–30^.

TBI is traditionally categorized using Glasgow Coma Scale (GCS), a clinical assessment of consciousness, or Abbreviated Injury Score (AIS), a pathoanatomical trauma score based primarily on computer tomography (CT) findings^12,31–36^. Both GCS and AIS (head) are independent predictors of mortality after TBI^31^. Many studies evaluating HRQOL after TBI use GCS to classify TBI^4^, and the vast majority of TBI-patients presenting to hospital are categorized as “mild”, defined as GCS of 13–15^32,37^. However, this will only consider consciousness and not incorporate structural injuries as seen on CT scans. Some studies suggest that using an anatomical injury assessment may increase the predictive capacity^14,18,36,38^.

The aim of this study was to explore the long-term HRQOL and risk of depression in trauma patients. We hypothesised that (1) trauma patients with a TBI have a reduced HRQOL and increased risk of depression compared to trauma patients without, and that AIS (head), contrast to GCS, can help predict HRQOL and risk of depression in mild TBI.

## Methods

### Study design

The study was based on a cohort treated for trauma at Södersjukhuset, Sweden, January–December 2019. The hospital is a Level II trauma center, amongst several in Stockholm, serving approximately 700 000 inhabitants. The city has also a level I trauma center with neurosurgical capabilities, and patients with moderate-severe TBI or polytrauma are typically referred there. Relevant baseline data prospectively assembled at the time of admission was retrospectively screened. Follow-up 18 to 24 months after admission was performed by mail. The research protocol was approved by the Swedish Ethical Review Authority (Dnr: 2019-06122). Written informed consent was obtained from all subjects or their next of kin prior to enrolment. All methods were performed in accordance with the relevant guidelines and regulations.

### Population

The Swedish Trauma registry (SweTrau) was used to identify the trauma population. SweTrau, a national trauma registry established in 2011, is based on the revised Utstein Trauma Template^39^. Inclusion criteria for the current study was age ≥ 18 years at the time of injury and a trauma alert activated at hospital or a New Injury Severity Score (NISS) of more than 15. Exclusion criteria were trauma alert activated without underlying trauma, non-traumatic injuries such as asphyxia and hypothermia, death prior to arrival, trauma more than 24 h prior of admission, foreign residents only temporarily visiting Sweden, or missing significant data (e.g., GCS at admission, AIS, inability to follow-up etc.). In case of multiple admissions during the set timeframe, only the first was included. TBI was identified through the International Classification of Disease, 10th version (ICD-10). All intracranial lesions and skull fractures were classified as TBI (supplementary table 1). The patients were then divided into TBI or NTBI. The TBI-cohort was divided into mild TBI (AIS head ≤ 2) and severe TBI (AIS head ≥ 3) Comorbidities was scored with American Society of Anesthesiologists (ASA) physical status and dichotomized into none/mild systemic disease (ASA-score ≤ 2) and severe systemic disease (ASA- score ≥ 3).

### Data collection

Demographic data was collected from medical records. GCS at admission was assessed by the attending clinician upon hospital admission. Scoring of AIS and ASA were performed by accredited AIS-scoring professionals according to the stated guidelines. Socioeconomic data was obtained by a questionnaire sent together with the HRQoL-questionnaires.

### Outcome

Two surveys were used to capture the patient’s subjective HRQoL: The RAND-36 and EQ-5D-3L. Both instruments are widely used in TBI-research, with high reliability and validity even with cognitive impairment^4,15,17^.

#### RAND-36

The RAND 36-item health survey is a generic self-administered HRQoL questionnaire developed by the RAND Corporation^40,41^. RAND-36 is a license-free equivalent to the commercial questionnaire *36-item short form* questionnaire (SF-36) validated in over 180 languages, including Swedish^40–42^. The survey entails 36 items, assessing eight domains^4,5,17,40,41^. The 5-item *general health dimension* (GH) measures overall health, the 10-item *physical functioning dimension* (PF) measures health limitations due to physical function, the 4-item *physical role functioning dimension* (RP) measures how physical health affects daily activities and work, the 2-item *bodily pain dimension* (BP) measures the intensity and effect pain has on health, the 4-item *vitality dimension* (VT) measures the energy level and fatigue, the 2-item *social functioning dimension* (SF) measures the effect of health on social activities, the 3-item *emotional role functioning dimension* (RE) measures how emotional health affects daily activities and work, the 5-item *mental health dimension* (MH) measures general mood, anxiety and depression, and one item measures development over time. All items were then analysed, and every dimension scored 0 to 100, where a higher number indicates a higher quality of life. The Swedish translation developed by the Swedish PROM-network was used in this study (RAND-36 version 1.02), which has a been proven to be equivalent to the English version^42^. Age-adjusted normal values for the Swedish population were derived from Ohlsson-Nevo et al. 2021^43^.

#### EQ-5D-3L

The 5-dimensional questionnaire EQ-5D was developed from the original in 1991 by the EroQol Group. It consists of a self-questionnaire assessing five dimensions (mobility, self-care, usual activities, pain/discomfort, and anxiety/depression) and three severity levels (no, moderate, and severe problems), along with a visual analogue scale. A higher number indicates higher HRQoL. The validated Swedish translation of EQ-5D-3L was used^44^, but not the visual scale due to technical limitations. Values for the Swedish general populations were obtained from Burström et. al (2014)^44^ and Björk et. al (1999)^45^.

#### Mood/depression

Mood, including signs of depression, was assessed with Montgomery Åsberg depression Rating Scale self-report (MADRS-S)^46^. It includes nine depression-related items that are rated on a 6-level scale, with 0 = minimal, 6 = maximal and a total of 0–54 points. Up to 12 points constitutes no or very mild depression, 13–19 points mild depression, 20–34 points moderate depression and > 34 points severe depression^47^.

### Statistical analysis

All data was analysed using R (Cran Project, v.4.0.4, Austria) through the visual interface R-studio (v. 1.4.1103, PBC, USA, now renamed “Posit”). Normal distribution was assessed with Shapiro–Wilk test. The results are presented as median with interquartile range for continuous data, and n (%) for nominal data, if not stated otherwise. Baseline characteristics among TBI and NTBI patients were assessed using the Mann–Whitney U test for quantitative variables, chi-squared test for categorical variables with expected count of at least 5, and Fischer’s exact test for categorical variables with expected count of less than 5. The significance level was set to 0.05. Ordered logistic regression was used to identify the most important subscales in SF-36, EQ-5D-3L and total MADRS-S score, with respect to differentiating patients with TBI or NTBI, AIS (head), and ASA-score. Both univariable and multivariable models were used. The multivariable models corrected for age, ASA (dichotomous) and AIS (dichotomous), except when already included in the model. The Bonferroni correction post hoc test was applied to account for multiple testing.

### Ethics approval

The research protocol was approved by the Swedish Ethical Review Authority (Dnr: 2019-06122).

## Results

### Patient demographics

**Figure 1:**
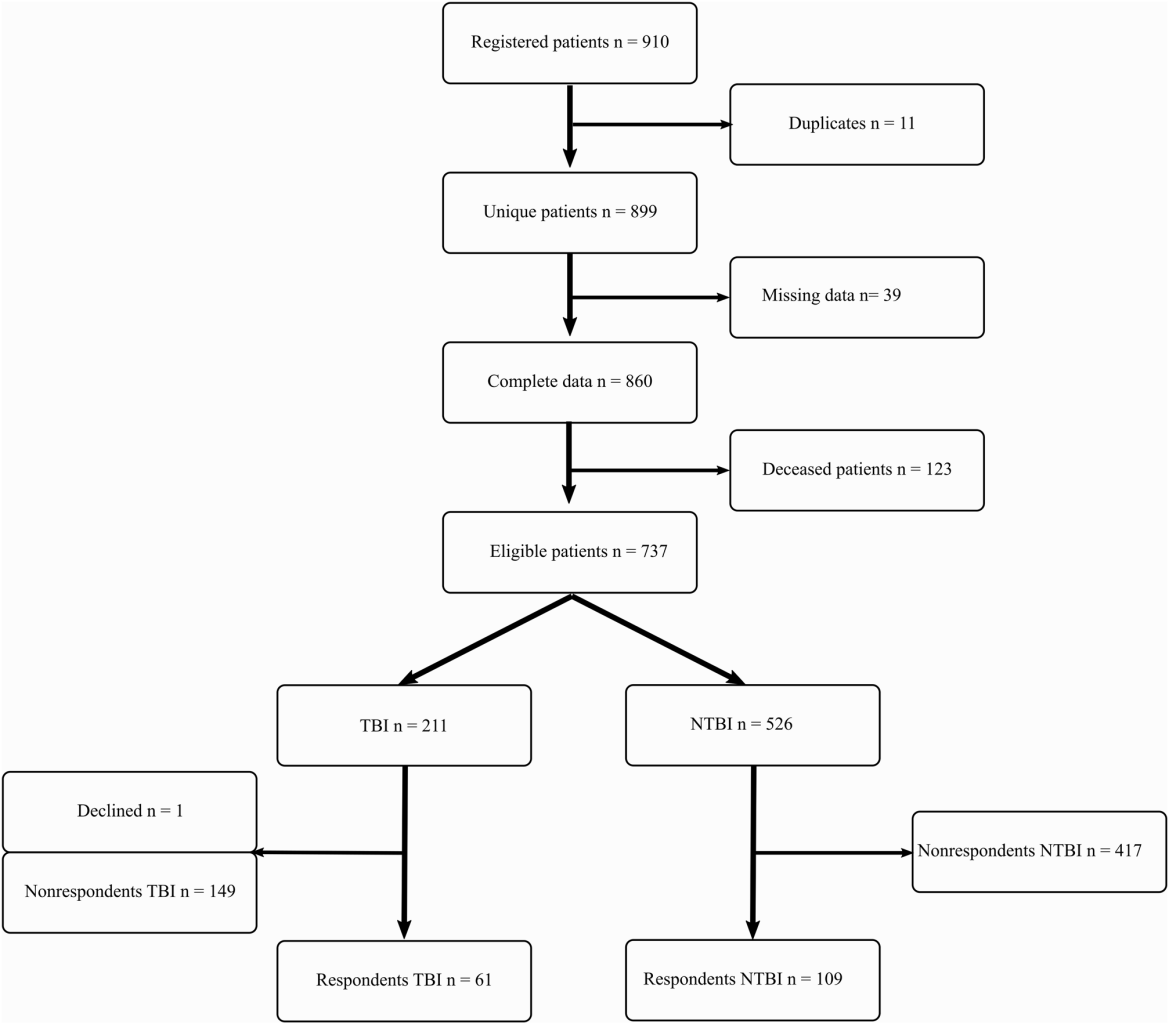
Enrolment in the study. A total of 11 patients were admitted more than once. Missing data includes foreign nationals not available for follow-up (n = 19) or missing contact details (n = 20).

Inclusion flow-chart is visualized in Fig. 1 and selected sociodemographic characteristics in Table 1. During the study period, 910 hospital visits that met the inclusion criteria were logged and eleven patients were admitted more than once. A total of 39 patients were excluded due to missing crucial data. At the time of follow-up, 123 of the remaining 860 patients were deceased. In the end, 61 TBI patients (median age 67, 44% female) and 109 NTBI patients (median age 63 50% female) were recruited. Falls, both low and high energy, were the main mechanism of injury in both groups, followed by bicycle accidents in the TBI-group and motor vehicle accidents (MVA) in the NTBI group. All but three patients had a GCS score of 15. The TBI population had more severe head injuries, whereas the NTBI population had more severe injuries to the lower extremities.

**Table 1:** Demography and social data of included patients with complete data, eligible for follow-up.

	TBI n = 61	NTBI n = 109	p-value
Gender, Female	27 (44.3%)	54 (49.5%)	0.5
Age (years)	67 (60, 76)	63 (49, 74)	**0.024**
ASA			0.5
1. Healthy	20 (32.8%)	41 (37.6%)	
2. Mild systemic disease	21 (34.4%)	37 (33.9%)	
3. Severe systemic disease	20 (32.8%)	31 (28.4%)	
Injury intention			0.2
Accident	57 (93.4%)	105 (96.3%)	
Self-inflicted	0 (0%)	2 (1.8%)	
Assault	4 (6.6%)	2 (1.8%)	
Mechanism of injury			0.06
Traffic-car	0 (0%)	16 (14.7%)	
Traffic-motorcycle	1 (1.6%)	4 (3.7%)	
Traffic-bicycle	10 (16.4%)	10 (9.2%)	
Traffic pedestrian	2 (3.3%)	4 (3.7%)	
Stabbing	0 (0%)	4 (3.7%)	
Blunt object	4 (6.6%)	6 (5.5%)	
Low energy fall	23 (37.7%)	33 (30.1%)	
High energy fall	21 (34.4%)	31 (28.4%)	
Other	0 (0%)	1 (0.9%)	
GCS			0.9
13–15 Mild	60 (98.4%)	107 (98.2%)	
9–12 Moderate	1 (1.6%)	0 (0%)	
3–8 Severe	0 (0%)	2 (1.8%)	
NISS	11 (6, 17)	9 (3, 14)	< 0.001**
AIS ≥ 3			
Head	33 (79%)	2 (4.1%)	< 0.001**
Face	1 (2.4%)	0 (0%)	0.5
Neck	0 (0%)	0 (0%)	NA
Thorax	4 (9.5%)	20 (41%)	**0.002**
Abdomen	1 (2.4%)	4 (8.2%)	0.4
Spine	2 (4.8%)	6 (12%)	0.3
Upper extremities	0 (0%)	0 (0%)	NA
Lower extremities	1 (2.4%)	17 (35%)	< 0.001**
Other	0 (0%)	0 (0%)	NA
Hospital days	3 (2, 4)	3 (1, 5)	0.60
GOS at discharge			0.3
3. Severe disability	12 (19.7%)	31 (28.4%)	
4. Moderate disability	48 (787%)	52 (46.7%)	
5. Good recovery	1 (1.6%)	26 (23.9%)	
Time to survey (months)	23.0 (20.7, 25,8)	23.9 (21.3, 26.3)	0.3
Retired	41 (67%)	48 (44.0%)	**0.004**
Highest education level			0.5
Compulsory school (grade 9)	14 (23.0%)	27 (24.7%)	
Upper secondary school (grade 12)	24 (39.3%)	32 (29.4%)	
College or university	23 (37.7%)	48 (44.0%)	
Ethnicity			0.7
Sweden	55 (90.2%)	92 (84.4%)	
Scandinavia	3 (4.9%)	5 (4.6%)	
Europe	1 (1.6%)	2 (1.8%)	
Outside of Europe	2 (3.3%)	9 (8.3%)	
Missing data	0 (0%)	1 (0.9%)	
Financial problems	7 (11.5%)	22 (20.2%)	0.2
Missing data	2 (3.3%)	1 (0.9%)	

Results expressed in median and (IQR) as well as numeric values and (%).

*ASA* American Society of Anesthesiologists Classification, *GCS* Glasgow Coma Scale, *NISS* New Injury Severity Score, *AIS* Abbreviated Injury Scale, *GOS* Glasgow Outcome Score.

Significant values are in bold.

*Significant after Bonferroni correction.

### Health related quality of life

**Figure 2:**
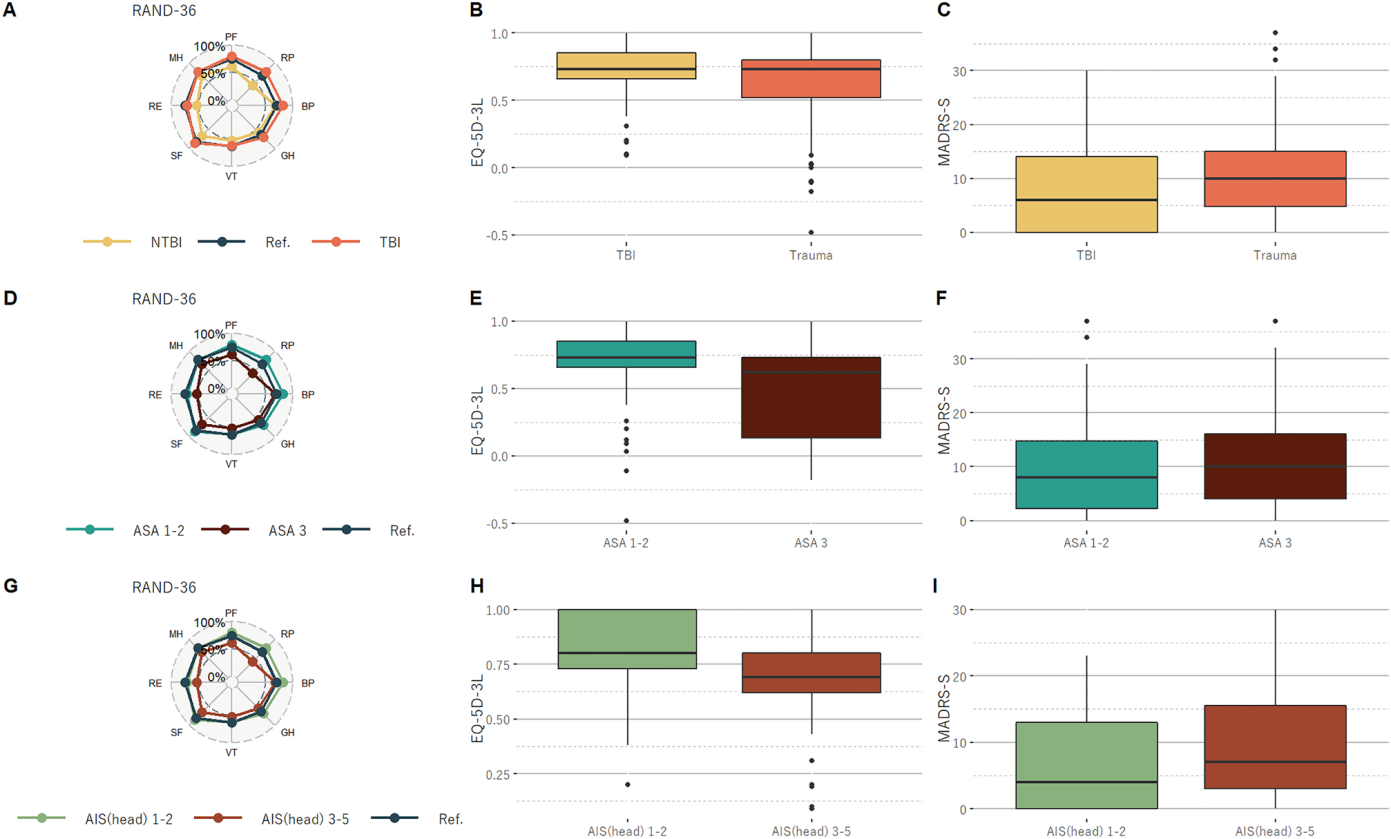
Health-related quality of life (HRQoL) assessed with RAND-36 (**A,D,G**) and EQ-5D-3L (**B,E,H**), and symptoms of depression assessed with Montgomery Åsberg depression Rating Scale self-report (MADRS-S) (**C,F,I**), comparing trauma patients with a concomitant traumatic brain injury (TBI) to trauma patients without (NTBI) (**A,B,C**), no or few comorbidities (ASA-score 1–2) to severe systemic morbidity (ASA-score 3) (**D,E,F**), and TBI severity defined by Abbreviated Injury Severity (AIS) head score of 3 (**G,H,I**).

**Figure 3:**
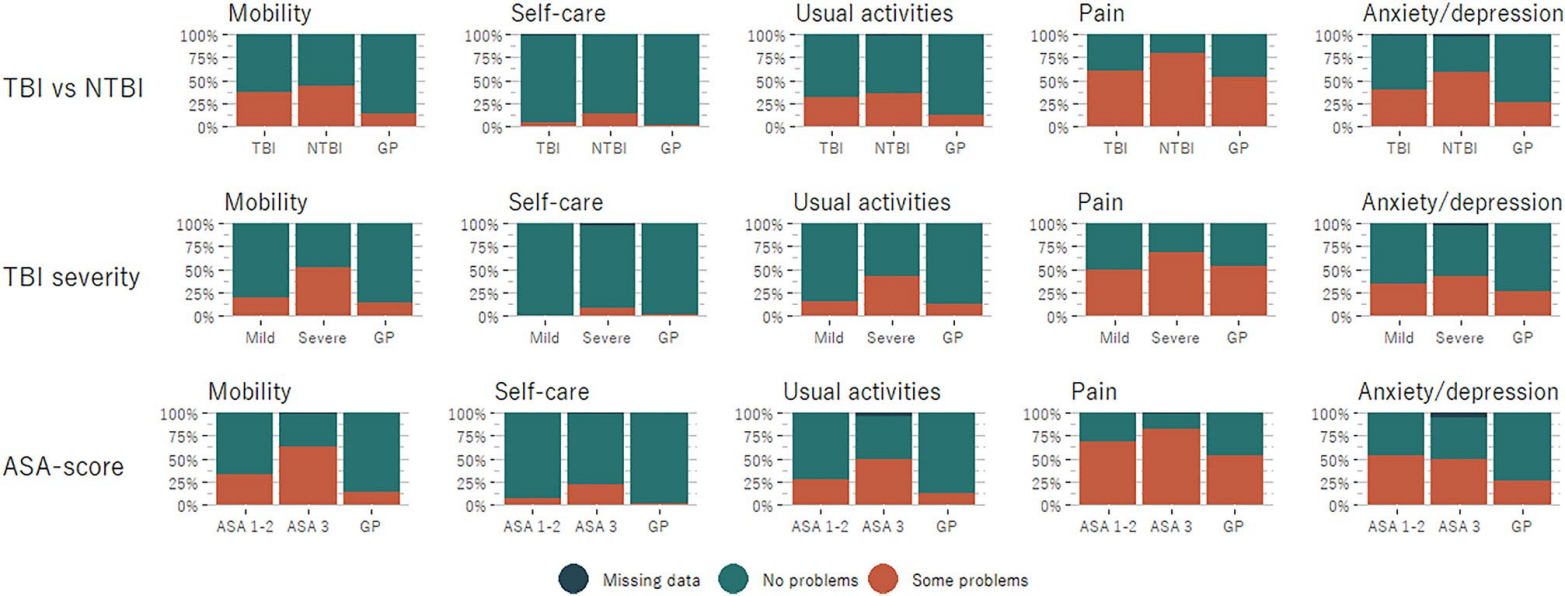
Problems reported by EQ-5D-3L dimension dichotomized into “no problems” and “some problems”. Comparing trauma patients with a concomitant traumatic brain injury (TBI) to trauma patients without (NTBI), TBI severity defined by Abbreviated Injury Severity (AIS) head score of 3, and no or few comorbidities (ASA-score 1–2) to severe systemic morbidity (ASA-score 3).

The results of the HRQoL assessments are listed in Tables 2, 3 and 4 and visualized in Figs. 2 and 3. TBI was associated with higher scores in all domains of RAND-36 compared to NTBI. After the post hoc test, the score for physical functioning and physical role functioning were significantly higher in the TBI-group (p < 0.001). TBI was also associated with higher scores in EQ-5D, but none were significant after the post-hoc test. An AIS (head) of three or higher was associated with lower scores in all domains of RAND-36 and EQ-5D in the TBI-group, however, none were significant after the post-hoc test. An ASA-score of three was also associated with lower scores in all domains of both RAND-36 and EQ-5D in both groups. Physical functioning and mobility were significant after the post-hoc test, as well as the EQ-5D-3L total scores. Socio-economy did not significantly affect outcome.

**Table 2:** Results TBI versus NTBI.

	TBI, N = 61^1^	NTBI, N = 109^1^	p-value^2^	p-value (multivariable)^2^	GP^3^
RAND-36					
Physical Functioning	81 (45, 95)	75 (45, 95)	0.2	< 0.001*	80 (73, 85)
Missing data	0	0			
Physical Role functioning	75 (0, 100)	25 (0, 100)	**0.031**	< 0.001*	76 (65, 77)
Missing data	0	0			
Bodily Pain	90 (45, 100)	62 (35, 90)	**0.012**	0.2	71 (70, 72)
Missing data	1	1			
Vitality	55 (40, 75)	50 (30, 70)	0.053	**0.006**	61 (59, 67)
Missing data	2	0			
Social role Functioning	88 (60, 100)	75 (50, 100)	0.058	0.2	84 (82, 86)
Missing data	1	2			
Emotional Role functioning	67 (33, 100)	33 (0, 100)	0.079	0.10	77 (77, 82)
Missing data	3	0			
Mental Health	80 (54, 88)	64 (45, 80)	**0.018**	**0.004**	77 (75, 80)
Missing data	2	0			
General Health	65 (45, 82)	55 (35, 75)	**0.022**	** < 0.001**	67 (65, 67)
Missing data	2	0			
EQ-5D-3L	0.70 (0.66, 0.85)	0.69 (0.52, 0.80)	**0.038**	** < 0.001**	0.92
Missing data	13	11			
Mobility			0.3	**0.005**	
1. No problems	38 (62%)	60 (56%)			86%
2. Some problems	23 (38%)	44 (41%)			14%
3. Extreme problems	0 (0%)	4 (3.7%)			0%
Missing data	0	1			
Self-care			0.066	0.8	
1. No problems	57 (95%)	92 (85%)			99%
2. Some problems	3 (5.0%)	15 (14%)			2%
3. Extreme problems	0 (0%)	1 (0.9%)			0%
Missing data	1	1			
Usual activities			0.6	**0.040**	
1. No problems	42 (69%)	68 (64%)			89%
2. Some problems	15 (25%)	36 (34%)			8%
3. Extreme problems	4 (6.6%)	3 (2.8%)			4%
Missing data	0	2			
Pain/discomfort			**0.004**	**0.001**	
1. No problems	24 (39%)	21 (19%)			46%
2. Some problems	33 (54%)	72 (67%)			50%
3. Extreme problems	4 (6.6%)	15 (14%)			4%
Missing data	0	1			
Anxiety/depression			**0.014**	**0.033**	
1. No problems	36 (60%)	42 (40%)			73%
2. Some problems	22 (37%)	59 (56%)			23%
3. Extreme problems	2 (3.3%)	5 (4.7%)			3%
Missing data	1	3			
MADRS-S	6 (0, 14)	10 (5, 15)	**0.034**	**0.008**	
Missing data	0	1			

Comparing health-related quality of life (HRQoL), assessed with RAND-36 and EQ-5D, and symptoms of depression assessed with Montgomery Åsberg depression Rating Scale self-report (MADRS-S), in trauma patients with a concomitant traumatic brain injury (TBI) to trauma patients without (NTBI) and the general population (GP).

1. n (%); Median (IQR), 2. Mann Whitney U test; Proportional odds ratio comparing TBI and NTBI. 3. Age adjusted population values.

Significant values are in bold.

*Significant after Bonferroni correction.

**Table 3:** Results AIS in TBI.

	AIS 1–2, n = 26^1^	AIS 3–5, n = 35^1^	p-value^2^	p-value (multivariable)^2^
RAND-36				
Physical Functioning	95 (69, 100)	65 (40, 95)	**0.009**	0.062
Missing	0	0		
Physical Role functioning	100 (56, 100)	25 (0, 100)	**0.002**	**0.008**
Missing	0	0		
Bodily Pain	95 (66, 100)	78 (45, 100)	0.5	0.4
Missing	0	1		
Vitality	72 (45, 85)	55 (40, 65)	0.088	0.12
Missing	0	2		
Social role Functioning	100 (69, 100)	75 (25, 100)	0.8	0.9
Missing	0	1		
Emotional Role functioning	67 (33, 100)	33 (0, 100)	0.9	0.9
Missing	3	0		
Mental Health	80 (54, 88)	64 (45, 80)	0.073	0.083
Missing	2	0		
General Health	65 (45, 82)	55 (35, 75)	**0.036**	0.12
Missing	2	0		
EQ-5D-3L	0.73 (0.69, 0.80)	0.69 (0.60, 0.75)	**0.004**	**0.013**
Missing	10	3		
Mobility			**0.013**	0.051
1. No problems	21 (81%)	17 (49%)		
2. Some problems	5 (19%)	18 (51%)		
3. Extreme problems	0 (0%)	0 (0%)		
Missing data	0	0		
Self-care			0.8	0.8
1. No problems	26 (100%)	31 (91%)		
2. Some problems	0 (0%)	3 (8.8%)		
3. Extreme problems	0 (0%)	0 (0%)		
Missing data	0	1		
Usual activities			**0.021**	0.057
1. No problems	22 (85%)	20 (57%)		
2. Some problems	4 (15%)	11 (31%)		
3. Extreme problems	0 (0%)	4 (11%)		
Missing data	0	0		
Pain/discomfort			0.13	0.2
1. No problems	13 (50%)	11 (31%)		
2. Some problems	12 (46%)	21 (60%)		
3. Extreme problems	1 (3.8%)	3 (8.6%)		
Missing data	0	1		
Anxiety/depression			0.5	0.5
1. No problems	17 (65%)	19 (56%)		
2. Some problems	8 (31%)	14 (41%)		
3. Extreme problems	1 (3.8%)	1 (2.9%)		
Missing data	0	1		
MADRS-S	4 (0, 13)	7 (3, 16)	0.09	0.10

Comparing health-related quality of life (HRQoL), assessed with RAND-36 and EQ-5D, and symptoms of depression assessed with Montgomery Åsberg depression Rating Scale self-report (MADRS-S), in TBI patients with AIS head 1–2 to TBI patients with AIS head 3–5.

1. n (%); Median (IQR), 2. Ordered logit.

Significant values are in bold.

*Significant after Bonferroni correction.

**Table 4:** Results ASA.

	ASA 1–2, n = 119^1^	ASA 3–4, n = 51^1^	p-value^2^	p-value (multivariable)^2^
RAND-36				
Physical Functioning	85 (58, 95)	45 (15, 80)	< 0.001*	**0.004**
Missing	0	0		
Physical Role functioning	67 (0, 100)	0 (0, 50)	** < 0.001**	**0.045**
Missing	0	0		
Bodily Pain	78 (45, 100)	54 (33, 78)	0.2	0.7
Missing	1	1		
Vitality	55 (40, 75)	45 (20, 60)	**0.001**	** < 0.001**
Missing	2	0		
Social role Functioning	88 (63, 100)	63 (25, 94)	0.9	0.7
Missing	1	0		
Emotional Role functioning	67 (0, 100)	33 (0, 100)	**0.028**	0.056
Missing	2	1		
Mental Health	68 (52, 85)	64 (44, 84)	0.12	**0.023**
Missing	2	0		
General Health	65 (45, 80)	45 (30, 62)	** < 0.001**	**0.013**
Missing	2	0		
EQ-5D-3L	0.73 (0.66, 0.80)	0.61 (0.11, 0.73)	< 0.001*	** < 0.001**
Missing	21	3		
Mobility			< 0.001*	0.13
1. No problems	80 (67%)	18 (36%)		
2. Some problems	38 (32%)	29 (58%)		
3. Extreme problems	1 (0.8%)	3 (6.0%)		
Missing data	0	1		
Self-care			**0.006**	0.4
1. No problems	110 (93%)	39 (78%)		
2. Some problems	8 (6.8%)	10 (20%)		
3. Extreme problems	0 (0%)	1 (2.0%)		
Missing data	1	1		
Usual activities			**0.002**	**0.004**
1. No problems	86 (72%)	24 (49%)		
2. Some problems	31 (26%)	20 (41%)		
3. Extreme problems	2 (1.7%)	5 (10%)		
Missing data	0	2		
Pain/discomfort			**0.030**	**0.041**
1. No problems	37 (31%)	8 (16%)		
2. Some problems	71 (60%)	34 (68%)		
3. Extreme problems	11 (9.2%)	8 (16%)		
Missing data	0	1		
Anxiety/depression			> 0.9	0.3
1. No problems	55 (47%)	23 (48%)		
2. Some problems	59 (50%)	22 (46%)		
3. Extreme problems	4 (3.4%)	3 (6.2%)		
Missing data	1	3		
MADRS-S	8 (2, 15)	10 (4, 16)	0.5	0.3
Missing	1	0		

Comparing health-related quality of life (HRQoL), assessed with RAND-36 and EQ-5D, and symptoms of depression assessed with Montgomery Åsberg depression Rating Scale self-report (MADRS-S), in TBI patients with ASA 1–2 to TBI patients with ASA 3.

1. n (%); Median (IQR), 2. Ordered logit.

Significant values are in bold.

*Significant after Bonferroni correction.

### MADRS-S

Higher score on MADRS-S was seen in the NTBI group, in the TBI-group with an AIS (head) of 3 or higher, and with an ASA-score of 3, but none were significantly different between the cohorts (Tables 2, 3 and 4, Fig. 2).

## Discussion

This present study found that patients having sustained trauma without TBI rated their HRQoL lower than patients with TBI. To our knowledge, this is the first study to compare a mixed severity TBI and NTBI cohort in this manner. We also investigated what factors were associated with HRQoL and depressive symptoms. We defined TBI with ICD-10 criteria and assessed the impact of type of trauma (TBI vs. NTBI), TBI-severity, age, gender, injury severity, and comorbidities, on HRQoL 2 years after the trauma. Our study included all patients admitted to Södersjukhuset, a level II trauma center located in Stockholm. The city also has a level I trauma center with neurosurgical care, and patients with moderate-severe TBI or polytrauma are typically referred there. In other words, any moderate-severe TBI or polytrauma included in this study were, according to local directives, in the wrong hospital. This is, however, the reality and is therefore of high clinical relevance, as level of trauma center may affect the outcome^48–50^.

The study found that, unexpectedly, NTBI patients had a slightly lower HRQoL compared to TBI patients, contradicting previous research^14,19,51^. TBI patients in the study had HRQoL values similar to the general population in Sweden, while NTBI patients scored lower, particularly in the domains of pain. One possible explanation for this could be that the TBI cohort in our study had a high median age and many were retired, which may have made subtle changes that are common after TBI, such as diminished concentration, executive functions, processing speed, and attention span^52^, less noticeable and therefore not affecting HRQoL as much. The NTBI cohort, on the other hand, had more injuries to the lower extremities which may have caused post-traumatic pain to a larger extent, with an impact on HRQoL. Another possible explanation is that the difference in HRQoL between TBI and NTBI seen in previous research is more specific to more severe trauma, and not in the mild-moderate range seen in our population. Mild TBI might, as discussed above, not have a severe impact on HRQoL as mild extremity trauma, compared to more severe TBI. A third possible explanation for this finding is impaired awareness or insight in patients with TBI which can potentially influence the HRQoL ratings. Patients with TBI may have difficulty understanding or completing the survey due to cognitive or communication impairments, which can lead to inaccurate or incomplete responses. We added the possibility for a next-of-kin to fill in the survey int the patient’s stead, in order to limit this. Only one entry was filled out this way. Additionally, patients with impaired awareness or insight may not fully understand the impact of their injury on their quality of life, but if patients are not aware of the full extent of their disability and it does not bother them, their HRQoL may be higher than external observers may assume. It is important to note that symptoms do not necessarily reflect HRQoL, and this highlights the importance of using patient-reported outcomes measures, such as HRQoL, in clinical decision-making to provide hope for both healthcare professionals and next of kin.

Furthermore, the study found that TBI severity measured with AIS (head) was inversely associated with HRQoL in the TBI-group, particularly in physical domains. The association between TBI severity and HRQoL is also disputed. Born et al.^14^, for example, did not find any association between TBI severity and HRQoL, except for AIS (head) of 5. A possible reason to our different findings is that previous studies usually pool mild TBI with no TBI, and as our findings suggest that patients with no TBI have a lower HRQoL, the difference between TBI severities might be overlooked. We used the ICD-10 codes for defining TBI within our predefined SweTrau cohort, due to its universal usage and clinical relevance for ongoing care. In our cohort, no patient in the TBI group had an AIS (head) of 0, and the 24 patients in the NTBI group who had an AIS (head) = 1 had these due to minor skin abrasions on the skull. In other words, by using the ICD-10 as inclusion criterion, the TBI-group included all minor intracranial lesions, e.g., concussion, but not the minor extracranial lesions of the head included in the AIS (head) definition. Born, on the other hand, dichotomized TBI into severe (AIS head > 2) and no/mild TBI. This emphasizes how symptoms, injury severity, and HRQoL are not the same.

Age was associated only with physical and role function of RAND-36 (data not shown). The prognostic role of age as a predictor of mortality is well studied^53–55^, but its role in HRQoL is under debate. Age is in some studies correlated to diminished HRQoL^12,56–59^, but not in others^13,60,61^. The conflicting results, and our weak association, warrants cation when assuming HRQoL based on age alone. Gender is another variable with conflicting results. Many studies show a poorer HRQoL in females compared to males^8,12,56,62,63^, but again, some do not^13,64^, and gender was not associated with any outcome in our study. Injury severity, defined by NISS, was not associated to any outcome in our cohort. Even though some studies have found a correlation between injury severity and HRQoL^8,12,13^, many do not^14–16,19,59^.

An interesting finding from this study was the association between comorbidity assessed with ASA-score, and HRQoL. The study found that an ASA-score of three was significantly associated with lower HRQoL in almost all aspects. The ASA-score is primarily used to assess patients preoperatively, and despite some limitations, such as inter-observer variability, it is still considered an important tool for risk assessment^65^. ASA-score has previously been shown to be a good approximation of comorbidities in trauma patients^66^, and an independent predictor of mortality after trauma^53,55^. However, the correlation between ASA-score and HRQoL is less studied. Both Ringburg et al.^63^ and Scholten et al.^12^ showed a decreased HRQoL after trauma in patients with comorbidity, but the definition of comorbidity was not further specified. We could not find any other studies addressing this issue, despite a rigorous literature search. Despite a rigorous literature search, we could not find any other studies addressing this issue. The relationship between ASA-score and HRQoL could have implications for early patient stratification and resource allocation in trauma care, and further research is needed to explore this relationship in more detail.

Previous studies have confirmed a correlation TBI and depression and other psychiatric conditions such as post-traumatic stress disorder (PTSD) and general anxiety disorder (GAD^21–25^, as well as between trauma and depression^28–30^. However, our study did not find any association between depression, assessed with the MADRS-S, and any of the parameters analyzed. One potential explanation for this discrepancy may lie, once again, in the high median age of our study population. Research suggests that the relationship between TBI and depression decreases with increasing age, with rates as low as 2.6–4.8% among the elderly population^22,25,67^.

This study has several limitations that should be taken into consideration when interpreting the results. Firstly, due to a response rate of 20–30%, there is a risk of selection bias. Compared to the eligible population, the patients included in our study were older and had higher ASA-score, NISS, and GCS (supplementary tables 2 and 3). The risk of underreporting by TBI-patients, due to cognitive limitations, may aggravate any selection bias compared to NTBI. There may also be a selection if patients reporting less pronounced depressive symptoms compared to non-responders. This could be caused by a self-selection process where patients without depressive symptoms may be more likely to participate in the study, leading to an underestimation of the prevalence of depressive symptoms in the overall population. It is overall difficult to determine how these differences may have affected our results^68^, so caution should be exercised when extrapolating our findings to a broader trauma population. Additionally, this cohort represents the severe end of the mild TBI spectrum due to the inclusion criteria of the SweTrau study, so our results should primarily be applied to "complicated" mild TBI patients with head CT findings. Another limitation of the study is the lack of baseline HRQoL assessments, which would be important in comparing ASA scores to HRQoL metrics, as comorbidities may have affected quality of life prior to the injury. In this study, we used generic instruments to measure HRQoL to compare the different groups. RAND-36 and EQ-5D are well-validated for TBI and trauma^4,69^, but TBI-specific instruments may be more sensitive for subtle cognitive changes post-TBI and should be considered as a supplement in future studies. The timing of long-term outcome measurement is still under debate. We chose the 2-year endpoint for our analysis as previous research has shown the biggest improvement within the first 12 months post-trauma a longer follow-up studies struggle with decreasing participation and increasing risk for confounders^12,56^.

This study has several strengths that make it an important contribution to the field. Firstly, it is a prospective study that includes all patients admitted to a level II trauma center, ensuring a broad and representative sample. Additionally, it has a long follow-up period and a clinically relevant control group, as patients present to emergency departments as "trauma patients" regardless of their injury. Even though the findings of this study should be extrapolated with care due to the limitations stated above, they raise several important questions and clinical implications. Firstly, it would be valuable to investigate why TBI-patients had such a high HRQoL, as this contradicts previous studies. Future studies based on TBI-specific surveys, and studies exploring the correlation between post-concussion symptoms and impact on HRQoL are needed. Secondly, it would be interesting to investigate how shifting demographics in trauma cohorts may impact outcome predictions. There is increasing evidence of a shifting demographics with increasing age of TBI-patients, in line with this study, which poses new challenges for the management of TBI, including risk of treatment bias^58,70–72^. Thirdly, it would be valuable to investigate the relationship between injury severity, symptoms, and HRQoL, as this study found that they are not always directly related, and further studies are needed to understand how they interact. Lastly, the study suggests that patients with severe systemic disease may be at a higher risk for poorer outcomes after TBI, and may require closer monitoring and more intensive treatment and follow-up. This highlights the importance of assessing and managing comorbidities in patients with TBI, as they may have a significant impact on recovery and long-term outcomes.

## Conclusion

The study found that two years after trauma, patients without a concomitant (TBI) reported a lower HRQoL than TBI patients, which has not been studied before. Additionally, the study found that increased TBI severity, measured with AIS (head), was associated with diminished HRQoL. The study also found that comorbidities, assessed ASA-score, were an important predictive factor of HRQoL, independent of injury severity. These findings suggest that individual factors prior to injury, such as comorbidities, may play a crucial role in determining long-term HRQoL outcomes for patients who have experienced trauma. Further research is needed to fully explore the correlation between ASA-score, TBI, and long-term HRQoL, to gain a more comprehensive understanding of the factors that may impact the recovery and well-being of trauma patients.

## Supplementary Information

Supplementary Table 1.

Supplementary Table 2.

Supplementary Table 3.

## Data Availability

The data that support the findings of this study are available on request from the corresponding author OK. The data are not publicly available due to them containing information that could compromise research participant privacy.
