# Supplementary material for: Long-term health-related quality of life after trauma with and without traumatic brain injury: a prospective cohort study
Source: Sci Rep. 2023 Feb 20;13:2986. doi: 10.1038/s41598-023-30082-4 (PMC9941121; doi:10.1038/s41598-023-30082-4)
Supplement: Supplementary file 1 — Supplementary Table 1. [file 41598_2023_30082_MOESM1_ESM.pdf]

Supplementary table 1: ICD-10 definition of TBI

**Table 1. ICD-10 codes defining TBI**	
S02.0	Fracture of vault of skull
S02.1	Fracture of base of skull
S02.3	Fracture of orbital floor
S02.7	Multiple fractures involving skull and facial bones
S02.8	Fractures of other skull and facial bones
S02.9	Fracture of skull and facial bones, part unspecified
S06.0	Concussion
S06.1	Traumatic cerebral oedema
S06.2	Diffuse brain injury
S06.3	Focal brain injury
S06.4	Epidural haemorrhage
S06.5	Traumatic subdural haemorrhage
S06.6	Traumatic subarachnoid haemorrhage
S06.7	Intracranial injury with prolonged coma
S06.8	Other intracranial injuries
S06.9	Intracranial injury, unspecified

Supplementary table 1. Table of ICD-10 codes defining TBI. All intracranial lesions and fractures of the skull are included. Facial fractures and fractures of the cervical spine are excluded.
