# Supplementary material for: Long-term health-related quality of life after trauma with and without traumatic brain injury: a prospective cohort study
Source: Sci Rep. 2023 Feb 20;13:2986. doi: 10.1038/s41598-023-30082-4 (PMC9941121; doi:10.1038/s41598-023-30082-4)
Supplement: Supplementary file 2 — Supplementary Table 2. [file 41598_2023_30082_MOESM2_ESM.pdf]

Supplementary table 2 : Responders vs nonresponders TBI

**Supplementary table Demography TBI**	**Resp n = 61 (28.9%)**	**Non-resp n = 150 (71.1%)**	**p-value**
**Gender, Female**	27 (44%)	52 (35%)	0.2
**Age (years)**	67 (60, 76)	56 (39, 73)	**<0.001***
**ASA**			0.4
1. Healthy	20 (33%)	61 (41%)	
2. Mild systemic disease	21 (34%)	45 (30%)	
3. Severe systemic disease	20 (33%)	43 (29%)	
4. Severe systemic disease constant threat to life	0 (0%)	1 (0.7%)	
**Injury intention**			0.7
Accident	57 (93%)	134 (89%)	
Self-inflicted	0 (0%)	1 (0.7%)	
Assault	4 (6.6%)	15 (10%)	
**Mechanism of injury**			
Traffic -car	0 (0%)	1 (0.7%)	
Traffic -motorcycle	1 (1.6%)	3 (2.0%)	
Traffic- bicycle	10 (16%)	20 (13%)	
Traffic pedestrian	2 (3.3%)	3 (2.0%)	
Blunt object	4 (6.6%)	21 (14%)	
Low energy fall	23 (38%)	84 (56%)	
High energy fall	21 (34%)	18 (12%)	
**GCS**			**0.018**
13-15 Mild	60 (98%)	135 (90%)	
9-12 Moderate	1 (1.6%)	11 (7.3%)	
3-8 Severe	0 (0%)	4 (2.7%)	
**NISS**	11 (6, 17)	12 (6, 19)	0.6
**AIS (head)**			0.7
0. None	0 (0%)	3 (2.0%)	
1. Minor	2 (3.3%)	1 (0.7%)	
2. Moderate	24 (39%)	73 (49%)	
3. Serious	30 (49%)	49 (33%)	
4. Severe	2 (3.3%)	17 (11%)	
5. Critical	3 (4.9%)	7 (4.7%)	
**Hospital days**	3 (2, 4)	3 (2, 4)	>0.9
**GOS at discharge**			>0.9
3. Severe disability	12 (20%)	29 (19%)	
4. Moderate disability	48 (79%)	120 (80%)	
5. Good recovery	1 (1.6%)	1 (0.7%)	

Demography of eligible TBI-patients Results expressed in median and (IQR) as well as numeric values and (%).

***ASA**: American Society of Anesthesiologists Classification, **GCS**: Glasgow Coma Scale, **NISS**: New Injury Severity Score, AIS: Abbreviated Injury Scale, GOS: Glasgow Outcome Score.*
