# Supplementary material for: Long-term health-related quality of life after trauma with and without traumatic brain injury: a prospective cohort study
Source: Sci Rep. 2023 Feb 20;13:2986. doi: 10.1038/s41598-023-30082-4 (PMC9941121; doi:10.1038/s41598-023-30082-4)
Supplement: Supplementary file 3 — Supplementary Table 3. [file 41598_2023_30082_MOESM3_ESM.pdf]

Supplementary table 3 : Responders vs nonresponders NTBI

**Supplementary table Demography NTBI**	**Resp = 109 (20.7%)**	**Non-resp=417 (79.3%)**	**p-value**
**Gender, Female**	54 (50%)	169 (41%)	0.090
**Age (years)**	63 (49, 74)	51 (33, 70)	**<0.001***
**ASA**			**0.046**
1. Healthy	41 (38%)	197 (47%)	
2. Mild systemic disease	37 (34%)	133 (32%)	
3. Severe systemic disease	31 (28%)	86 (21%)	
4. Severe systemic disease constant threat to life	0 (0%)	1 (0.2%)	
**Injury intention**			**0.026**
Accident	105 (96%)	377 (90%)	
Self-inflicted	2 (1.8%)	5 (1.2%)	
Assault	2 (1.8%)	35 (8.4%)	
**Mechanism of injury**			
Traffic -car	16 (15%)	91 (22%)	
Traffic -motorcycle	4 (3.7%)	11 (2.6%)	
Traffic- bicycle	10 (9.2%)	27 (6.5%)	
Traffic pedestrian	4 (3.7%)	8 (1.9%)	
Traffic other	0 (0%)	2 (0.5%)	
Gunshot wound	0 (0%)	1 (0.2%)	
Stabbing	4 (3.7%)	27 (6.5%)	
Blunt object	6 (5.5%)	35 (8.4%)	
Low energy fall	33 (30%)	142 (34%)	
High energy fall	31 (28%)	72 (17%)	
Other	1 (0.9%)	1 (0.2%)	
**GCS**			0.8
13-15 Mild	107 (98%)	411 (99%)	
9-12 Moderate	0 (0%)	4 (1.0%)	
3-8 Severe	2 (1.8%)	2 (0.5%)	
**NISS**	9 (3, 14)	3 (2, 9)	**<0.001***
**AIS (head)**			0.3
0. None	86 (79%)	311 (75%)	
1. Minor	23 (21%)	104 (25%)	
2. Moderate	0 (0%)	1 (0.2%)	
3. Serious	0 (0%)	1 (0.2%)	
**Hospital days**	3 (1, 5)	1 (1, 3)	
**GOS at discharge**			**<0.001***
3. Severe disability	31 (28%)	86 (21%)	
4. Moderate disability	52 (48%)	138 (33%)	
5. Good recovery	26 (24%)	193 (46%)	

Demography of eligible TBI-patients Results expressed in median and (IQR) as well as numeric values and (%).
***ASA**: American Society of Anesthesiologists Classification, **GCS**: Glasgow Coma Scale, **NISS**: New Injury Severity Score, AIS: Abbreviated Injury Scale, GOS: Glasgow Outcome Score*
